# Finite element analyses of lateral condyle fracture fixation in paediatrics regarding configuration of Kirschner-wire

**DOI:** 10.1186/s12891-022-05897-3

**Published:** 2022-10-28

**Authors:** Sangbin Jeon, Wooyeol Ahn, Jongbeom Oh, Jaiwoo Chung, Junwon Choi, Soonchul Lee

**Affiliations:** 1 CHA Graduate School of Medicine, 120 Hyeryong-Ro, Pocheon, Gyeonggi-Do 11160 Republic of Korea; 2 Department of Orthopaedic Surgery, CHA University School of Medicine, 335 Pangyo-Ro, Bundang-Gu, Seongnam-Si, Gyeonggi-Do 13488 Republic of Korea; 3 Department of Molecular Science and Technology, Ajou University, Gyeonggi-Do, Suwon-Si, 16499 Republic of Korea

**Keywords:** Finite element analyses, Fixation, Kirschner-wire, Lateral condyle fracture

## Abstract

**Background:**

This study aimed to discover the most stable outcome among different Kirschner-wire (K-wire) configurations for fixation of a lateral condyle fracture (Milch type II) in different loads of stress by using finite element analyses (FEA).

**Methods:**

The right humerus of a 6-year-old boy with a lateral condyle fracture (Milch type II), was modelled with a computer aided engineering. Using FEA, peak von Mises stress and stiffness were evaluated first for a single K-wire fixation by varying the angle (0, 5, 10, 15, 20, 25, 30 degrees). Then, based on the single K-wire result, assessment of peak von Mises stress and stiffness were evaluated via FEA for two- or three-wire fixation under various configurations (two convergent, two parallel, three divergent).

**Results:**

Single K-wire fixation by 5 and 25 degrees had the lowest peak von Mises stress. The fracture site showed higher stiffness at 0, 5 and 15 degrees. Considering the collected results and clinical situation, 5 degree K-wire was selected for the FEA of multiple K-wire fixation. For multiple K-wire fixation, three divergent (5–20-35 degrees) K-wires showed better stability, both in peak von Mises stress and stiffness, than any two-K-wire configurations. Among two K-wire fixations, two divergent (5–50 degrees) K-wires provided the lowest von Mises stress in varus and valgus while two divergent (5–65 degrees) K-wires showed better results in flexion, extension, internal and external rotation, and both configurations showed similar results in stiffness.

**Conclusions:**

We successfully created a paediatric lateral condyle fracture (Milch type II) model which was used to conduct FEA on different K-wire configurations to achieve stability of the fracture. Our results show that an initial K-wire inserted at 5 degrees, followed by the insertion of a second divergent wire at either 45 or 60 degrees provides the most stability in two K-wire fixations in this type of fracture repair.

**Supplementary Information:**

The online version contains supplementary material available at 10.1186/s12891-022-05897-3.

## Background

Lateral condyle fracture of the distal humerus is the second most common elbow fracture in paediatrics, accounting for approximately 12 to 17% of entire elbow fractures, with a peak incidence for age 6 years [1–6]. Classifications of lateral condyle fractures were described for years, with the Milch and Jakob classifications preferred in clinical practice [7, 8]. The most common and unstable fracture pattern is a fracture that extends medially into the trochlear groove, which is known as Milch type II. Surgery using two or three Kirschner wires (K-wires) is the preferred treatment for lateral condylar fractures displaced more than 2 mm.

Although understanding of the biologics of lateral condyle fracture healing has recently increased, treatment of lateral condylar fractures is still associated with a high complication rate, including nonunion and malunion, regardless of the modality used [9]. Many studies have addressed ways to decrease the complication rate [4, 10–13], including various methods of reduction [13–18], types of fixation [19, 20] amount of casting, length of K-wire fixation [21–24], and whether the wires should be left out of (percutaneous K-wires) or buried under (subcutaneous K-wires) the skin.

However, most of the studies lack details such as the angle of the first wire when inserting multiple wires, the exit location of a wire, and the tendency of von Mises stress and stiffness according to pin configurations. To our knowledge, no study has assessed the configuration of K-wires in lateral condyle fracture fixation using finite element analyses (FEA). This study aimed to examine the most stable configuration of the K-wires according to the angle of the K-wires, the number of K-wires used, and the positions in which the K-wires were placed.

## Methods

This study was undertaken with the approval of the institutional review board of our institution’s Medical Ethics Committee. A geometric model of the lateral condyle fracture of the humerus was extracted from normal computed tomography (CT) images of the right elbow of a 6-year-old male child from our hospital’s picture archiving communications systems (PACS) format. The CT data was chosen because of its clear cartilage profile with no other pathological findings. The model went through an image process by Mimics Innovation Suite software, Version 20 (Materialize, Belgian). Due to the grayscale of CT images, the humerus, capitellum, cartilage, and growth plate were manually segmented according to the software’s protocol.

**Fig. 1:**
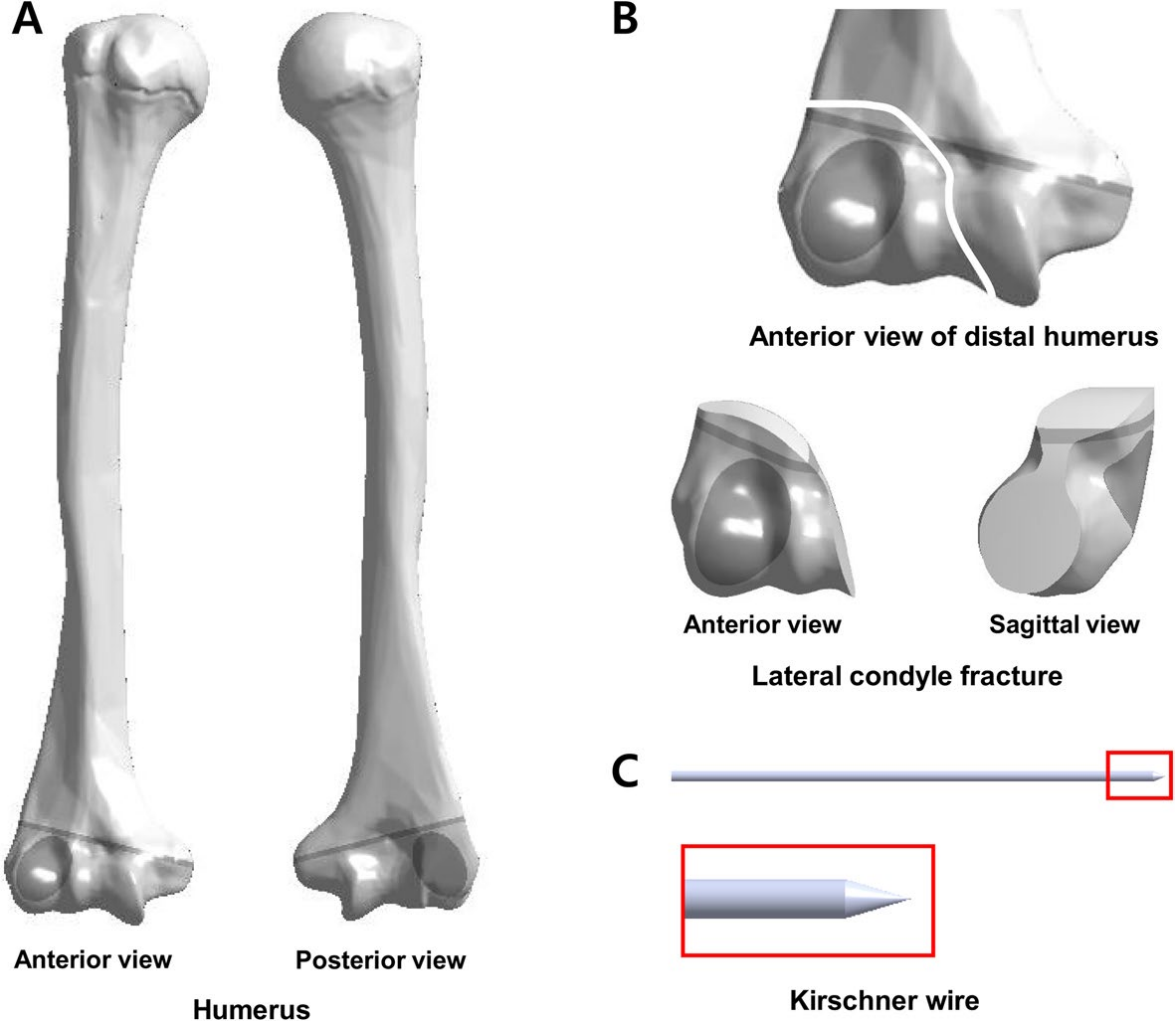
The geometrical distal humerus of a 6-year-old boy and Kirschner-wire utilised in this study

This processed image was sent to computer aided design software, SolidWorks 2018 edition (Dassaut Systemes-simula, France), and converted for adequate FEA (Fig. 1a). A typical Milch type II fracture model which is located above the level of the growth plate and extends into the trochlear groove at the middle of the olecranon fossa was processed by using the same software (Fig. 1b). Using the same provided engineering software, a geometric K-wire (1.6-mm diameter and 25-cm length) model was generated (Fig. 1c).

**Fig. 2:**
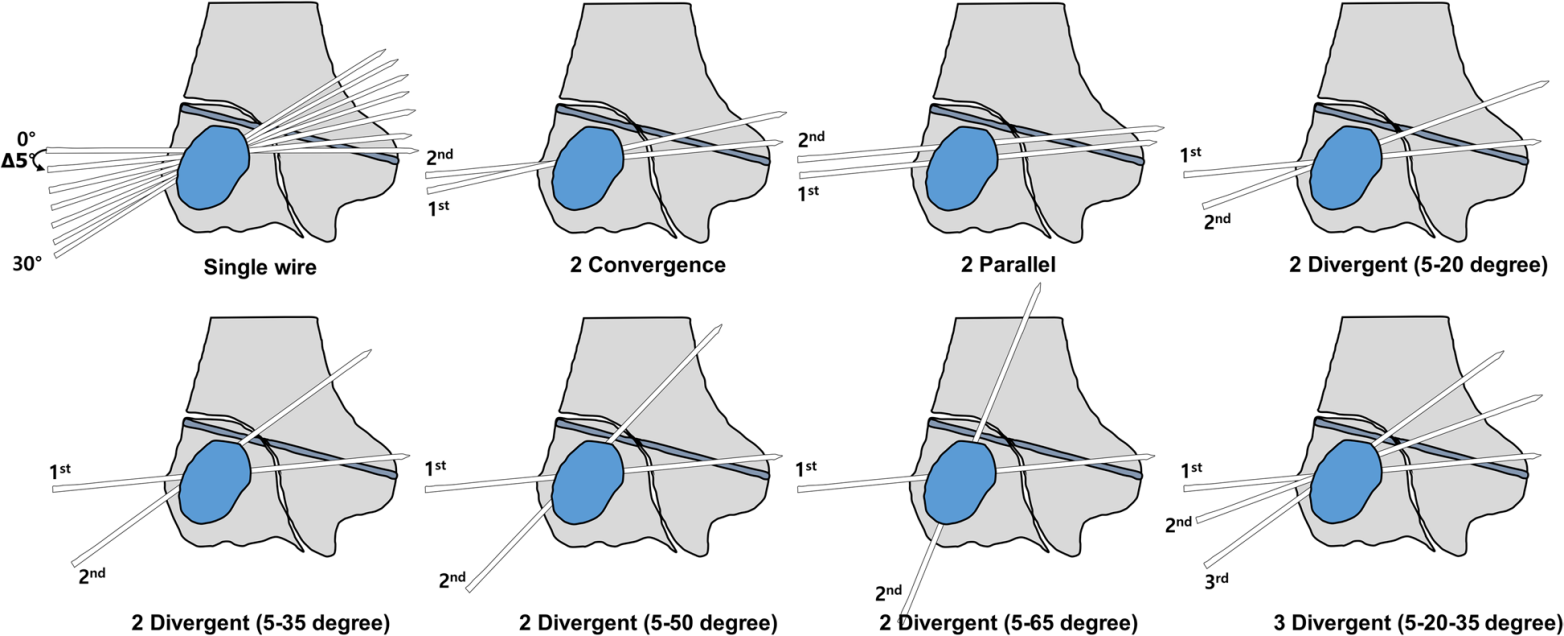
Schematics of the Kirschner-wire pinning of a lateral condyle fracture. 1^st^, 2^nd^, and 3^rd^ indicate the order of insertion, 1^st^ wire was set as a guide wire

Various models of K-wire insertion into the lateral condyle fracture were created. First, we created a model of a single K-wire through the fracture segment of the humerus to ascertain the most stable angle for K-wire placement. To achieve this, the fractured bony segment was aligned adjacent to the fracture line of the humerus. The joint line of the distal humerus was located, along with the X–Y plane. Then, the single K-wire was inserted at 0 degrees or perpendicular to the X–Y plane. We then increased the angle of the single K-wire in 5-degree increments from 0 to 30 degrees (Fig. 2).

Next, simulations were conducted in various multiple K-wire configurations as follows. A) Two convergent K-wires, in which two wires were crossed at the fracture site; B) Two parallel K-wires; C) Two divergent K-wires (5–20 degrees); D) Two divergent K-wires (5–35 degrees); E) Two divergent K-wires (5–50 degrees); F) Two divergent K-wires (5–65 degrees); and G) Three divergent K-wires (5–20-35 degrees). For each K-wire configuration, the first single K-wire was inserted as a guide, determined by the result of the single K-wire stability model, and the other wire was inserted at a specified angle to this guide (Fig. 2).

The finished 3-dimensional models were imported into Midas NFX, Version 2021 (MIDAS IT, South Korea) to perform the FEA. The material properties of each segment used in this study were summarised in Supplemental Table 1 [25–27]. Stainless steel was used to make the K-wires. All the assembled models were meshed and pre-processed. Tetrahedral 10-node elements were applied for precise analysis. A single node was selected as a reference point at the loading surface. The assembled models applied a load of 50 N along the reference axis. Translational motion was applied to the X, -X axis and Y, -Y axis to simulate flexion, extension, valgus, and varus stress. Equivalent load was applied as a torsional moment along the Z, -Z axis to simulate internal and external rotation.

**Table 1:** The number of nodes and elements in each segment

Segment	Number of elements	Number of nodes
Cortical bone	134,578	52,687
Cancellous bone	154,272	42,496
Growth plate	45,727	24,285
Cartilage	142,514	34,157
Capitellum	39,584	13,241
K-wire (Stainless steel)	4268	5124

We first analysed and documented the peak and distribution of von Mises stress of the single K-wire according to its angle at different loads. Also, stiffness of the fracture site was calculated for each load at different angles. The same procedures were performed in the multiple K-wires models, according to the different K-wire configurations.

## Results

The number of nodes and elements in each segment are listed in Table 1. The simulation results are presented as a contour representing images of the von Mises stress distribution for the different K-wire configurations (Supplemental Fig. 1).

### Single K-wire

**Fig. 3:**
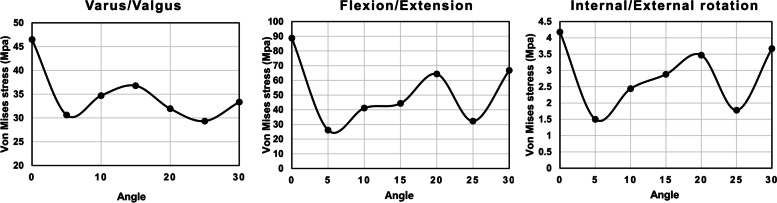
Variance of peak von Mises stress of a single Kirschner-wire according to different angles and loads

**Fig. 4:**
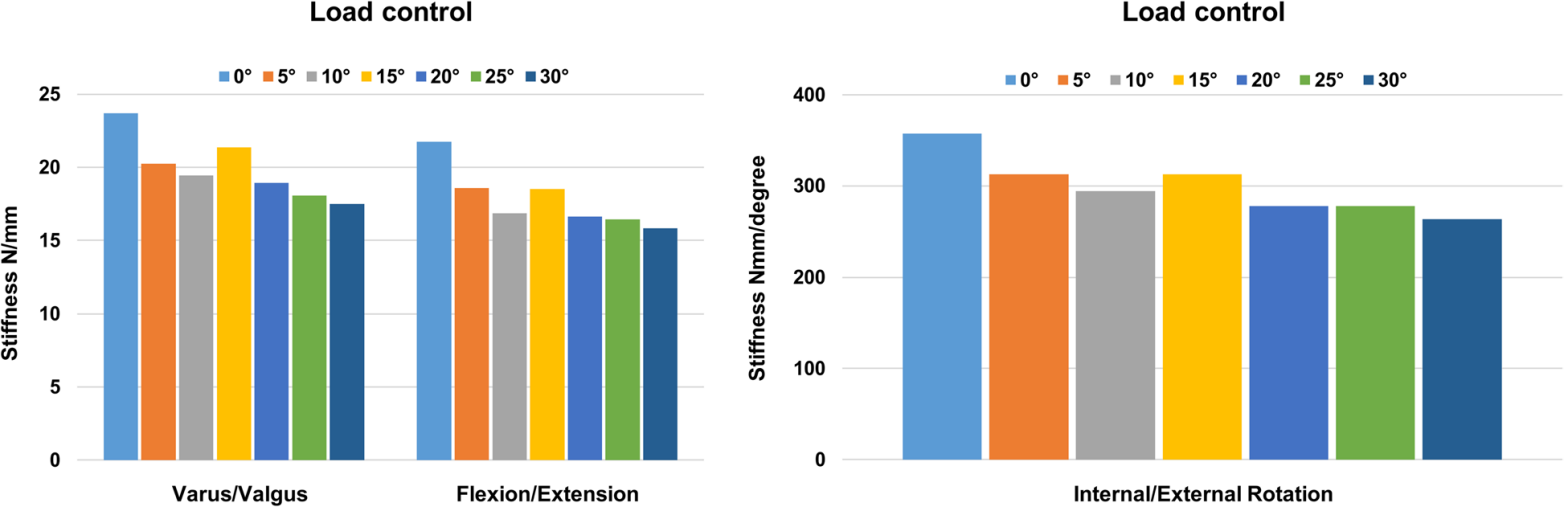
Stiffness of the fracture site after single Kirschner-wire fixation by varying angle and load

Different peak von Mises stress was observed in the single K-wire, depending on its angle of insertion, in flexion, extension, varus, valgus, and internal and external rotation (Fig. 3). The results showed that the peak von Mises stress differs in each load and angle of K-wire placement. However, similarities were observed in each load. Two minimum von Mises stresses were formed regardless of load at 5 and 25 degrees, respectively. Detailed peak von Mises stress regarding load can be seen in Supplemental Fig. 2. Peak von Mises stress was focused at the exit of the K-wire on the cartilage surface of the fracture segment. The result of stiffness of the single K-wire showed that 0, 5, and 15 degrees were more stable than any other degrees (Fig. 4). Among those, the K-wire fixed at 0 degrees demonstrated the best stiffness. A K-wire at a 5 degree angle was stiffer than one at 15 degrees in flexion, extension, internal and external rotation, while it was weaker in varus, and valgus.

### Multiple K-wires

Based on the results of the performed analyses of stability of the single K-wire at various angles, multiple K-wire configurations were assembled. A single K-wire was inserted at a 5 degree angle and was used as a guide for the location of the second or third K-wires. We compared the peak von Mises stress and stiffness at different K-wire configurations.

**Fig. 5:**
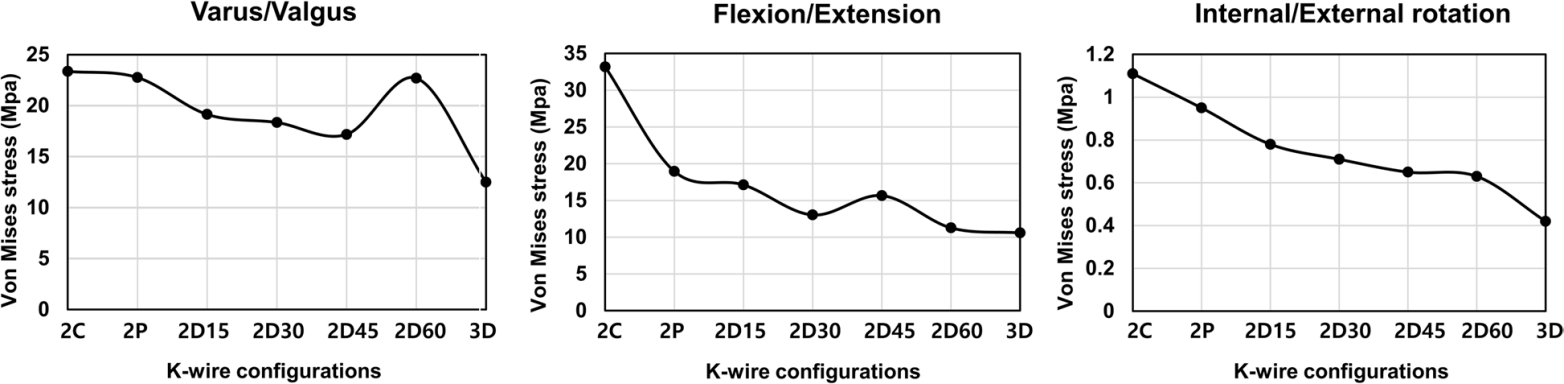
Variance of peak von Mises stress of the Kirschner-wire by multiple wire configurations according to different loads

**Fig. 6:**
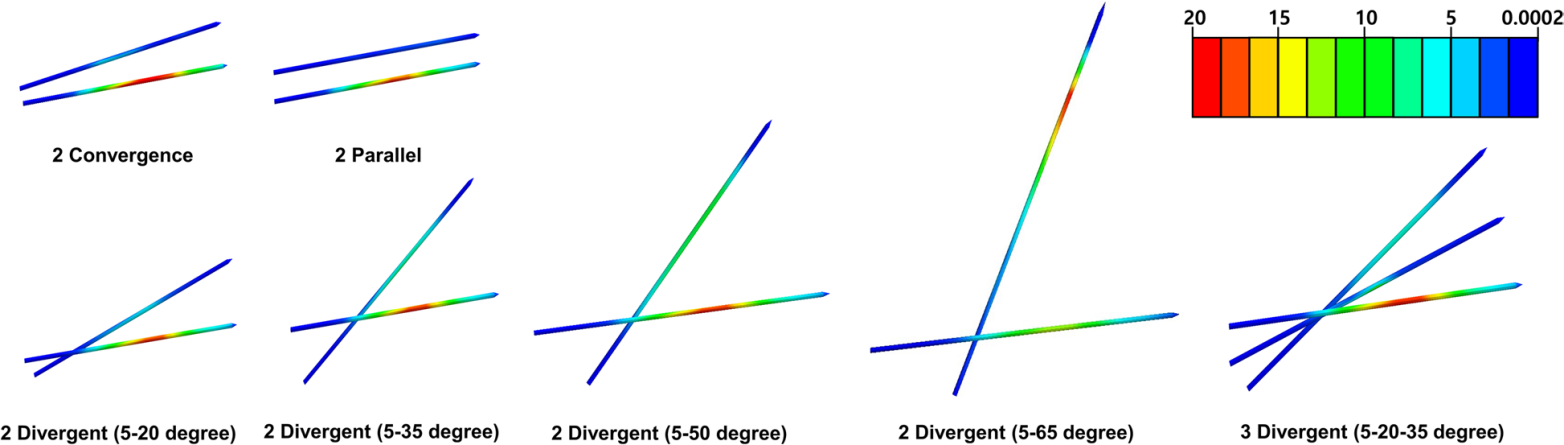
Stress distribution tendency of two and three Kirschner-wire configurations

**Fig. 7:**
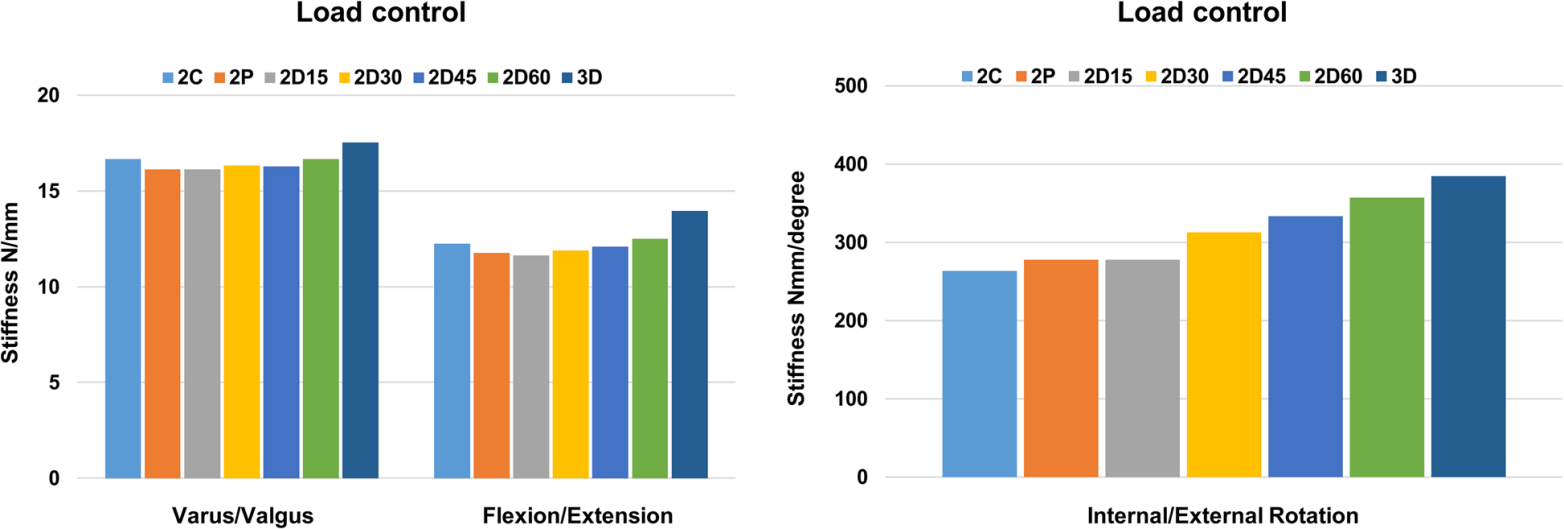
Stiffness of the fracture site after multiple Kirschner-wire fixations by varying loads. 2C: 2 Convergence. 2P: 2 Parallel. 2D15, 2D30, 2D45, 2D60: 2 Divergent (5–20 degree) (5–35 degree) (5–50 degree) (5–65 degree) in each. 3D: 3 Divergent (5–20-35 degree)

The results demonstrated that three divergent K-wire configuration was more stable than two-K-wire configurations, regardless of load type (Fig. 5). Detailed peak von Mises stress regarding load can be seen in Supplemental Fig. 3. Among the two K-wire configurations, the two divergent configurations showed lower peak von Mises stress compared to the two convergent and two parallel K-wire configurations. Among the two divergent configurations, two divergent (5–65 degrees) K-wires showed the smallest von Mises stress than the other divergent K-wire configurations in flexion, extension, internal and external rotation. However, a 45-degree total angle in the two divergent (5–50 degrees) K-wire configurations showed the smallest von Mises stress in varus and valgus load. The von Mises stress distribution of multiple K-wire configurations was also investigated (Fig. 6). The results demonstrated that among the two-K-wire configurations, more stress was induced in the second wire as the angle from the first wire increased. The investigation of stiffness showed that a three divergent (5–20-35 degrees) K-wire configuration was more stable than any two-K-wire configuration in every load. Among the two-K-wire configurations, two divergent K-wires at a 60 degree angle to each other showed the best stiffness (Fig. 7). The stress distribution of the fracture site peaked at the surface of the fracture border.

## Discussion

From the perspective of biomechanical testing, finite element modelling has several advantages over cadaver or animal studies as it allows the problems and variability that often affect human and animal models to be eliminated and/or controlled. Repeated investigations are allowed in FEA, and it provides continuous, quantitative, and accurate results of the stress imposed on bones. FEA is more applicable in paediatric fracture studies since paediatric clinical trials have more limitations than an adult study. Previous researchers have focused on K-wire configurations in paediatric supracondylar humerus fractures, both biomechanically and computationally [28–34]. Kamar et al. [34] investigated FEA of supracondylar fractures, but the study of lateral condyle fractures with FEA has not yet been performed. With regard to the research on lateral condyle fractures, a few studies were limited to biomechanical tests of K-wire configurations, and differing fixation materials such as screws or large K-wires [35]. Schlitz et al. [36] compared biomechanical analysis of fixation materials (K-wires versus screw) in lateral condyle fractures and Bloom et al. [37] reported biomechanical analyses in lateral condyle fractures in paediatrics.

We successfully created a lateral condyle fracture (Milch type II) model by using a 6-year-old male patient’s CT images and were able to determine optimal K-wire fixation through FEA. K-wire, which is widely used in clinical surgeries, was selected instead of screw fixation. First, we investigated the most stable angle of insertion for a single K-wire. The result revealed two minimum peak von Mises stress values of the K-wire at 5 degrees and 25 degrees according to loads applied in different directions. An insertion at 5 degrees was 13% and 23% more stable than a 25-degree insertion at varus/valgus and internal/external rotation, respectively. Meanwhile, the stiffness of the bone fracture site was the highest when a K-wire was inserted at 0 degrees. A K-wire at 0 degrees showed an average of a 13% better stiffness than a K-wire at 5 degrees at every load. However, this is not a big difference when considering that a 0-degree K-wire insertion has more unstable von Mises stress compared to a 5-degree insertion by 153%, 339%, and 276% at varus/valgus, flexion/extension, internal/external rotation, respectively. When considering the combination of peak von Mises stress of the K-wire and stiffness of the bone fracture site, the insertion of a single K-wire at 5 degrees was considered the best angle for stability.

Our results differed from the Arbeitsgemeinschaft für Osteosynthesefragen (AO) guidelines [38], which suggested that the first K-wire be inserted parallel to the joint surface, which is a 0-degree angle, and should penetrate the capitellum. However, in this study, the first K-wire was placed through the capitellum at an angle of 5 degrees since it showed the best stability in the 1 wire fixation and was transverse to the medial side and exited the humerus bone. Further analyses may be warranted in the future using a more realistic model.

Based on the single K-wire FEA result, diverse multiple K-wire configurations were simulated to find the most stable position. A three divergent (5–20-35 degrees) K-wire configuration showed better stability in peak von Mises stress at every load compared to that of a two-K-wire configuration. Among the two-K-wire configurations, two divergent (5–65 degrees) K-wires showed the lowest peak stress at the wire in flexion, extension, and internal and external rotation while two divergent (5–50 degrees) K-wire configuration showed the lowest peak stress imposed on the K-wire in varus, and valgus load. The peak von Mises stress distribution showed that the load of the obliquely inserted second wire increased according to its angle of insertion. This is because the larger difference between the angle of insertion of the two K-wires generates more distance between wires at the exit point, and results in stress concentration in a particular wire. Moreover, the peak von Mises stress of a particular K-wire was located at the exit site from the cortical bone. These trends were observed similarly in every load control. In general, three divergent (5–20-35 degrees) K-wires showed better stiffness compared to two K-wire configurations. Among the two K-wire configurations, an insertion of two divergent K-wires showed considerable stiffness compared to a convergent or parallel configuration. Among divergent wire configurations, an increased angle showed a direct correlation with stiffness. However, there was no remarkable difference between two divergent 45-degree (5–50 degrees) and two divergent 60-degree (5–65 degrees) configurations.

The results of peak von Mises stress and stiffness modelling suggested that a configuration of two divergent K-wires at 45 degrees to each other could be more beneficial than two divergent K-wires at 60 degrees in varus/valgus load. Moreover, a two divergent (5–50 degrees) K-wire configuration has more advantage in clinical outcomes. Cadaver investigations of two divergent 60-degree K-wire configurations showed that it failed to obtain bicortical purchase and bounced off the far cortex [39]. The tendency of bending and skidding over the opposite cortex was observed, resulting in the replacement of K-wire with a thicker fixation material [40]. Although 3 wire fixation showed the best stability, occasionally, fixation of 3 wires can encounter technical difficulty in the real clinical situation because 6 years old children have limited bone area. In this context, optimizing the fixation by using 2 wires would be reasonable.

A previous biomechanical study using a synthetic bone model [37] showed that two divergent 60-degree pins and three divergent pins were the most stable pin configurations for lateral condyle fracture repairs. Furthermore, they concluded that two divergent 60-degree K-wire showed better von Mises stress and stiffness compared to two divergent 30-degree K-wire. These conclusions generally correlate with our study results, although there were some differences noted. Our study differs from the previous research in that an investigation into the angle of insertion of the first single K-wire was performed, and an analysis of two divergent 45-degree (5–50) K-wire was also performed, where they were not performed previously. These additional experiments were possible due to the utilisation of FEA, which shortened the analysis time and effort.

There were some limitations to this study. First, the model generated of the lateral condyle fracture does not represent all types of lateral condyle fractures. However, we chose to focus on the most common type of fracture with the most prevalent age. Second, our research was only conducted in silico. The extracted model from the CT images may not perfectly correlate with real physical conditions because of the existence of complex surrounding tissues in human body. To overcome this problem, we referred to the similar loading and boundary condition suggested by the preceding [25–27, 34, 37]. The aim of this study lies in the investigation of trends rather than demonstrating the absolute values. Clinical trials are required to overcome the limitations of our study. Lastly, the precise motion of the load was not considered. As many muscles originate and insert in the elbow region, it was difficult to reflect the precise load from these muscles. Future studies are necessary for to elaborate on the bone model and reflect these limitations by using advanced software. Our results also require clinical confirmation to apply these findings to surgical practice for paediatric lateral condyle fractures.

## Conclusions

In conclusion, we successfully created a lateral condyle fracture (Milch type II) model and conducted a mechanical analysis, using FEA, of different K-wire configurations used to stabilise a lateral condyle fracture of the distal humerus. After fixation of the first K-wire at 5 degrees, three wires fixation showed the best stability and two divergent 45- or 60-degree K-wires appeared to provide the best stability among 2 wires configurations, with a selection of the degree of divergence being based on clinical status.

## Supplementary Information

**Additional file 1: Supplemental Table 1.** The material properties of each segment [25–27]. **Supplemental Fig. 1.** Representative images of the von Mises stress distribution for different Kirschner-wire configurations. **Supplemental Fig. 2.** Peak von Mises stress of a single Kirschner-wire after fixation by varying angle and load. **Supplemental Fig. 3.** Peak von Mises stress of the Kirschner-wire by multiple wire configurations according to different loads.

## Data Availability

The datasets used and/or analyzed during the current study are available from the corresponding author upon reasonable request.

## Abbreviations

K-wire: Kirschner-wire
FEA: Finite element analyses
CT: Computed tomography
C: Convergent
P: Parallel
D: Divergent
