# Supplementary material for: Finite element analyses of lateral condyle fracture fixation in paediatrics regarding configuration of Kirschner-wire
Source: BMC Musculoskelet Disord. 2022 Oct 28;23:940. doi: 10.1186/s12891-022-05897-3 (PMC9615206; doi:10.1186/s12891-022-05897-3)
Supplement: Supplementary file 1 — Additional file 1: Supplemental Table 1. The material properties of each segment [25–27]. Supplemental Fig. 1. Representative images of the von Mises stress distribution for different Kirschner-wire configurations. Supplemental Fig. 2. Peak von Mises stress of a single Kirschner-wire after fixation by varying angle and load. Supplemental Fig. 3. Peak von Mises stress of the Kirschner-wire by multiple wire configurations according to different loads. [file 12891_2022_5897_MOESM1_ESM.docx]

**Supplementary materials**

**Supplemental Table 1. The material properties of each segment [25-27]**

| **Segment** | **Elasticity (Gpa)** | **Poissons’s ratio (λ)** |
| --- | --- | --- |
| **Cortical bone** | 16.8 | 0.3 |
| **Cancellous bone** | 0.84 | 0.2 |
| **Growth plate** | 0.006 | 0.495 |
| **Cartilage** | 0.015 | 0.45 |
| **Capitellum** | 0.01 | 0.45 |
| **Stainless steel (K-wire)** | 2 | 0.3 |

**Supplemental Fig. 1. Representative images of the von Mises stress distribution for different Kirschner-wire configurations**

**
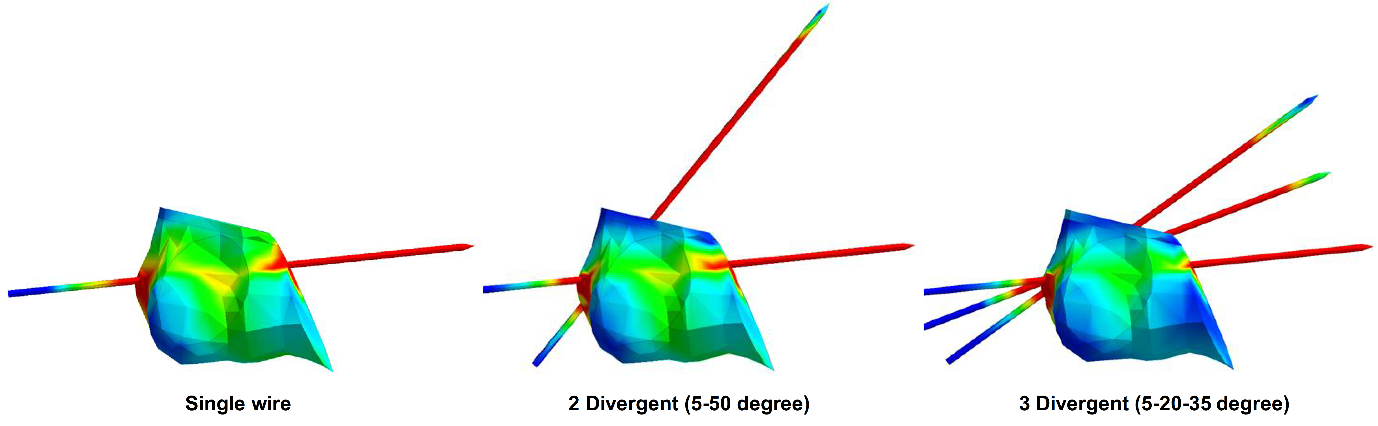
**

**Supplemental Fig.2. Peak von Mises stress of a single Kirschner-wire after fixation by varying angle and load**

**
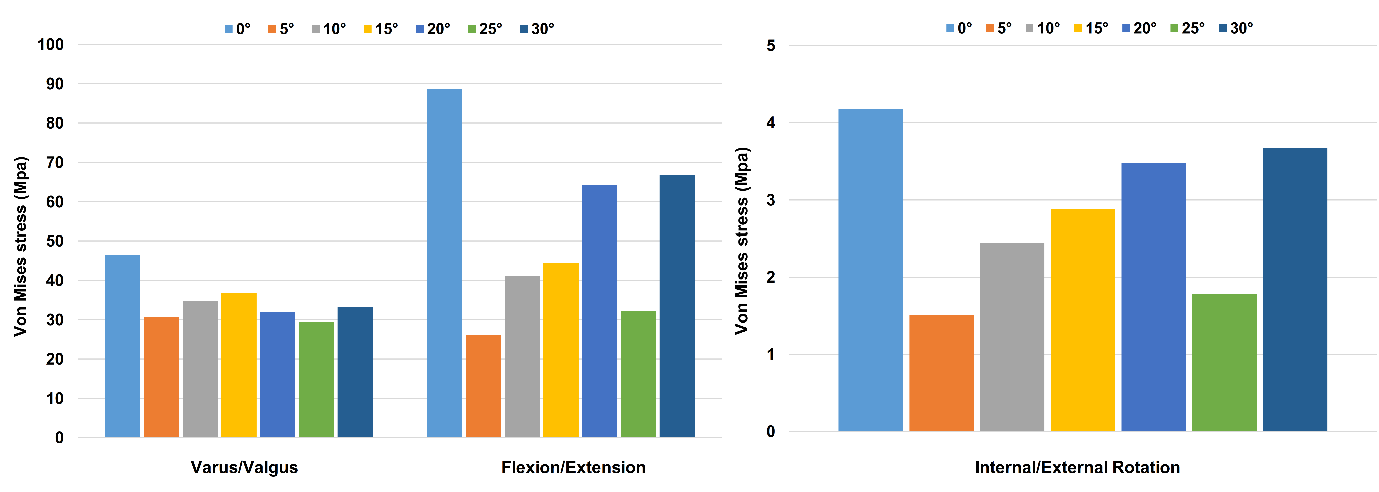
**

2C: 2 Convergence

2P: 2 Parallel

2D15, 2D30, 2D45, 2D60: 2 Divergent (5-20 degree) (5-35 degree) (5-50 degree) (5-65 degree) in each

3D: 3 Divergent (5-20-35 degree)

**Supplemental Fig. 3. Peak von Mises stress of the Kirschner-wire by multiple wire configurations according to different loads**

**
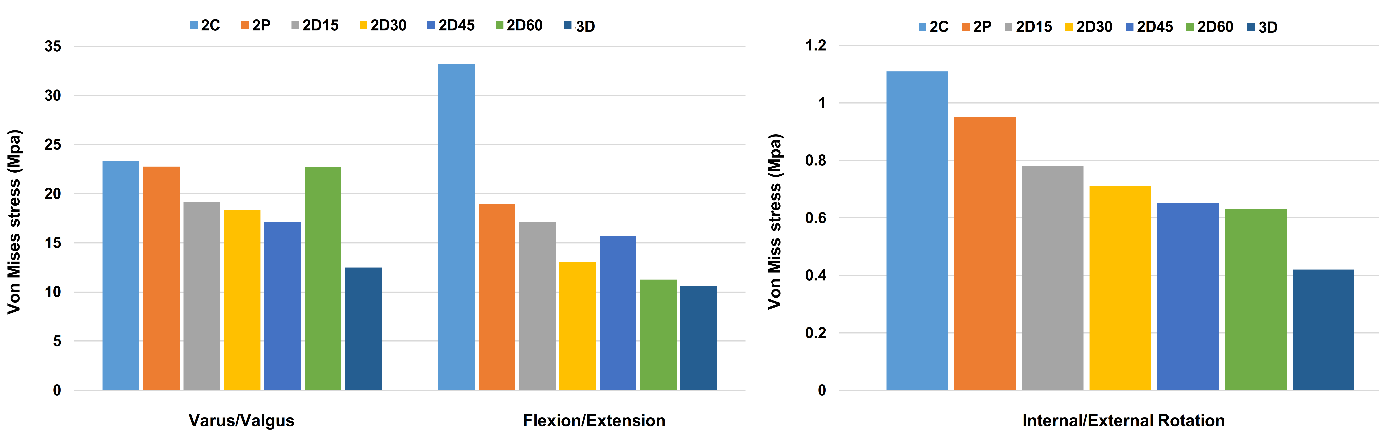
**

2C: 2 Convergence

2P: 2 Parallel

2D15, 2D30, 2D45, 2D60: 2 Divergent (5-20 degree) (5-35 degree) (5-50 degree) (5-65 degree) in each

3D: 3 Divergent (5-20-35 degree)
